# Mixing of Apples and Oranges in Milk Research: A Cohort Analysis of Non-Fermented Milk Intake and All-Cause Mortality

**DOI:** 10.3390/nu12051393

**Published:** 2020-05-13

**Authors:** Karl Michaëlsson, Liisa Byberg

**Affiliations:** Department of Surgical Sciences, Uppsala University, 75185 Uppsala, Sweden; liisa.byberg@surgsci.uu.se

**Keywords:** milk, dairy, mortality, cohort, fat, non-fermented

## Abstract

Mortality in relation to type of milk intake is unclear. We present mortality rates by intake of non-fermented milk fat content type and examine the degree of bias when other fat content types of non-fermented milk are kept in the reference category. For this purpose, we used a longitudinal cohort consisting of 61,433 women who had been administered food frequency questionnaires in 1987–1990 and in 1997, and analyzed time to death. Non-fermented milk consumption was divided into low ≤0.5%, medium 1.5%, or high fat 3%. For each specific type of milk, the first analysis (A) is restricted to those who consumed less than one serving per day of the other milk subtypes. In the second analysis (B), everyone is retained, i.e., leading to a reference category “contaminated” with other milk consumers. During follow-up, 22,391 women died. Highest (≥3 glasses/day) vs. lowest consumption category of milk (<1 glass/day) with 0.5% fat content was associated with a multivariable hazard ratio (HR) of 1.71 (95%CI 1.57–1.86) in analysis A, whereas the same comparison with a “contaminated” reference category in analysis B provided a HR of 1.34 (95%CI 1.24–1.45), *p*-value for homogeneity <0.0001. The corresponding HRs for 1.5% fat milk were: 1.82 (95%CI 1.63–2.04) and 1.38 (95%CI 1.25–1.51), and for 3% fat milk 1.95 (95%CI 1.77–2.15) and 1.40 (95%CI 1.29–1.52). HR for ≥3 glasses/day of total milk was 1.95 (95%CI 1.84–2.06). We observe a higher mortality in women with high milk consumption, irrespective of milk fat content. A “contaminated” reference group substantially attenuates the actual estimates.

## 1. Introduction

There is an ongoing debate about health benefits of dairy products, including non-fermented milk intake. For the ultimate clinically important outcome, time to death, long lasting interventional studies are not feasible. Cohort studies have provided mixed results, with some presenting lower, some null, and some higher rates of all-cause mortality with higher milk consumption [1]. Moreover, it is presently unclear whether only high consumption of whole milk is related to mortality or also low-fat milk consumption [2,3]. Potential explanations for the inconsistent findings may be related to diverse ranges of milk consumption in different populations but also to different analytical approaches. Specifically, comparisons between studies can be hampered, not only by non-similar higher range exposure categories but also by mixed reference categories. When separately examining skimmed or low fat milk and whole non-fermented milk intake in relation to mortality [2,3], whole milk consumers have been retained in, for example, the reference category of low-fat milk consumers. Therefore, the reference category consists of not only the intended group of low-consumers of low-fat milk but also of high consumers of whole milk, leading to a misclassification of this exposure category. This is like mixing apples (low consumption of low fat milk) with oranges (high consumption of whole milk). We hypothesize that such an analytical approach would lead to biased estimates due to “contamination” of the reference category. Therefore, we re-analyzed data from our cohort study [4], now with a longer follow-up, and present results by non-fermented milk fat content in relation to mortality. We further show the consequences of retaining or not retaining consumers of other non-fermented milk fat content types in the analysis.

## 2. Materials and Methods

We used a previously described [4] population-based longitudinal cohort, the Swedish Mammography Cohort (SMC), part of the national research infrastructure SIMPLER (www.simpler4health.se; Swedish Infrastructure for Medical Population-Based Life-Course and Environmental Research). The SMC started in 1987–1990 when 74% of all 90,303 women aged 39–74 years residing in two Swedish counties completed a questionnaire covering diet (food frequency questionnaire, FFQ) and lifestyle, enclosed with a mailed invitation to a routine mammography screening. In 1997, a subsequent expanded questionnaire was sent to the 56,030 still eligible women (response rate 70%). We excluded those with an implausible value for total energy intake [4]. In the present study, we included 61,433 women, without a prevalent cancer diagnosis at baseline in the SMC with information from 1987–1990, of whom 38,331 also had updated information from 1997. Using a valid and reproducible FFQ, the participants reported their average frequency of consumption of up to 96 foods and beverages during the past year, including non-fermented milk (low fat ≤0.5%, medium fat 1.5%, or high fat 3%), soured milk and yogurt, and cheese. Pasteurization of consumer sold milk in Sweden is mandatory since 1937. Few in Sweden use raw milk or ultra-heat treated (UHT) milk. Therefore, we were unable to compare risk estimates for raw or UHT milk with pasteurized milk. According to a validation study of the self-reported milk intake, the Spearman correlation between the FFQ and four 7-day food records every third month (a gold standard) was approximately 0.7 [5]. As the primary outcome, we considered all-cause mortality registered between baseline and September 2015 in the Swedish Total Population Register. The study has ethical approvals by the Regional Ethical Review Boards in Uppsala and Stockholm, Sweden.

**Figure 1:**
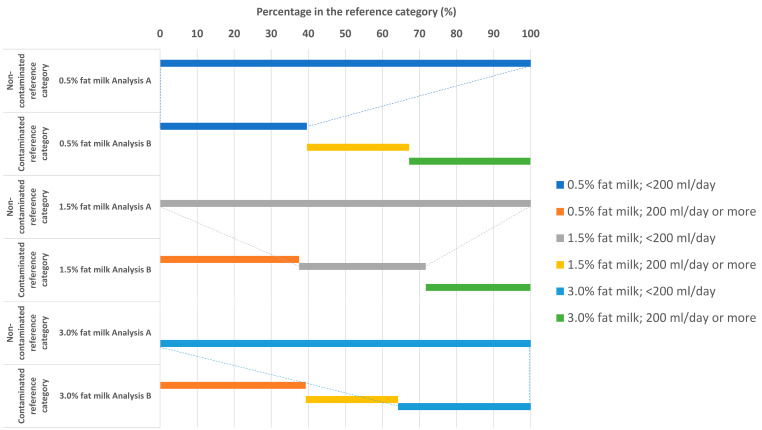
Proportion of different types of milk consumption by % fat in the reference category of analysis **A** (non-contaminated category) and analysis **B** (contaminated category). The dotted lines display that those in reference group of Analysis **A** are only a minority part of the reference group of Analysis **B**.

Since the denominator in the main analysis is time at risk, each participant accrued time at risk from the study entry until date of death or the end of the study period (30 September 2015). We calculated death rates, and age- and multivariable-adjusted hazard ratios (HRs) and their 95% confidence intervals (CIs), for categories of milk intake (<1, 1 to <2, 2 to <3, 3 or more glasses/day; with one glass of milk = 200 mL) by fat content (low ≤0.5%, medium 1.5%, and high 3%) with the exposure considered as time-updated cumulative averages, i.e., after the second questionnaire examination the mean consumption of the first and second reported consumption was used. For each specific fat content type of milk, the first analysis (A) is restricted to those who consume less than one serving per day of the other milk fat content types. In the second analysis (B), everyone is retained, i.e., leading to a reference category “contaminated” with consumers of other fat content types of non-fermented milk. The different exposures of the reference category are illustrated in Figure 1. Thus, in analysis B, the reference category of low fat (0.5%) milk consists of not only women with a low consumption of low fat milk (<200 mL/day) but also “high” consumers of other types of milk (both with a fat content of 1.5% and with a fat content of 3%). The analytical approach A avoids the problem of this misclassification.

Results for total non-fermented milk intake are also presented. The time-updated multivariable model included age, body mass index, height, total energy intake, total alcohol intake, healthy dietary pattern, calcium and vitamin D supplementation, ever use of cortisone, educational level, living alone, physical activity level estimated as metabolic equivalents, smoking status, estrogen replacement therapy, nulliparity, and weighted Charlson’s comorbidity index as described before [4]. We also present mean non-fermented milk intake by fat content in relation to education and marital status.

## 3. Results

The mean total intake of non-fermented milk at baseline in SMC was 240 mL per day. With increasing milk intake (Table 1), the energy intake was higher; however, there were small differences in the participants’ body stature, fermented milk intake, marital status, comorbidity, and educational level for the different categories of milk intake.

Using baseline data in the SMC, there are inverse correlations between the reported intakes of different non-fermented milk products. Thus, low fat (0.5%) milk intake has a negative correlation of −0.18 (*p* < 0.00001) with 1.5% fat milk and a negative correlation of −0.22 with 3% fat milk (*p* < 0.00001). Importantly, 45% of women consuming <1 glass reduced fat milk/day (0.5% or 1.5% fat) consumed 1 glass or more of 3% fat milk/day, and 64% of women consuming <1 glass 3% fat milk/day consumed 1 glass or more of reduced fat milk.

During a mean follow-up of 23 years (maximum 29 years), 22,521 women (total time at risk 1,438,676 person-years) died. In Table 2, we present the results for mortality by fat content type of non-fermented milk. Highest (≥3 glasses/day equivalent to 600 mL/day or more) vs. lowest consumption category of milk (<1 glass/day or <200 mL/day) with 0.5% fat content was associated with a multivariable HR of 1.71 (95% CI 1.57–1.86) in analysis A, whereas the same comparison with a “contaminated” reference category in analysis B provided a HR of 1.34 (95% CI 1.24–1.45), with a *p*-value for homogeneity <0.0001 of the estimates. The corresponding HRs for milk with 1.5% fat were 1.82 (95% CI 1.63–2.04) and 1.38 (95% CI 1.25–1.51), and for milk with 3% fat 1.95 (95% CI 1.77–2.15) and 1.40 (95% CI 1.29–1.52). HR for ≥3 glasses/day of total milk intake was 1.95 (95% CI 1.84–2.06). We found no heterogeneity between the estimates for the highest category of the different milk fat content types (*p* = 0.13). The attenuation of the estimates with a contaminated reference category was similar after adjustment for the intakes of the other milk fat subtypes (Table 2).

When different milk fat content types were considered as continuous variables (last column of Table 2), the relative differences between the non-contaminated and the contaminated HRs were even more pronounced. For example, the multivariable-adjusted HR of mortality was 1.12 (95% CI 1.10–1.14) per 200 mL higher consumption of 0.5% fat milk in analysis A whereas the corresponding HR in analysis B was 1.02 (95% CI 1.00–1.03).

Only small differences, 20 mL/day or less, in reported average milk intakes by fat content were found between categories of education and marital status (Table 3).

## 4. Discussion

Using our population-based cohort, we show that higher mortality with high milk consumption is observed irrespective of the milk fat content, results not previously presented. Moreover, we display that, using an analysis with a “contaminated” reference group when investigating specific milk fat categories, the associations are substantially weakened when compared to estimates using a proper comparison group. This is explained by the fact that the reference group in the contaminated analysis B consists of not only low consumption of the specific milk type under study (e.g., <200 mL/day of low fat milk) but also moderate to high consumption (≥200 mL/day) of medium and whole fat milk. Due to the negative correlation between consumption of different milk types, i.e., low consumers of low fat milk have an even higher likelihood of high consumption of high fat milk, the extent of misclassification is largest in the reference category. Adjustment for milk fat content [3] does not solve this issue. Drug testing in an efficacy clinical trial would not be designed such that the placebo pill was intended to be partially contaminated with the drug under study.

Our analysis was enabled by our sizable cohort with a large number of outcomes and with many women consuming high amounts of milk. In the Nurses’ Health Study and the Health Professionals’ Follow-up Study [3], the authors used less than one serving per week as a reference and 1.5 servings/day or more as highest exposure category for skimmed or low-fat milk, while the corresponding figures for whole milk was less than 1 serving per month and 2 servings per week or more. Despite the use of a contaminated reference category and a moderately high exposure in the analyses, slightly higher mortality rates were observed in the highest intake category. In contrast, in a large Japanese cohort study, high consumption was related to somewhat lower mortality rates, but the comparison was made between consumption of, on average, half a serving per day (100 mL) and never drinking milk [6]. Hence, there may be a U- or J-shaped pattern of mortality with non-fermented milk consumption and presenting such a pattern as a hazard ratio per unit change is not advisable, an approach commonly used in meta-analysis [1]. If the hazard ratio per unit change is based on a specific milk type by fat content while retaining other non-fermented milk consumption in the analysis, the result will be a severely biased calculation as displayed in the last column of Table 2. Those classified as low consumers of low fat milk will on average have an admixture of moderate to high consumption of milk with higher milk fat content, leading to a consequence of misclassification and attenuated hazard ratios. We furthermore display negative correlations between consumption of different types of milk and this pattern leads to differential misclassification of specific milk consumption by fat content and further attenuated estimates based on the contaminated analysis B. The true pattern of the risk curve from different studies cannot be resolved without a unified approach for the analysis and, preferentially, pooling of data or a federated data analysis.

We found only modest differences in milk consumption by socioeconomic aspects, previously suggested to explain associations between milk consumption and mortality [4,7]. Apparently, this is not a valid concern in our setting [7]. In contrast to Scandinavia, milk intake in the U.S. seems to be more strongly linked with socioeconomic status [8,9]. In the Nurses’ Health Study and the Health Professionals’ Follow-up Study [3], no adjustments had been made for socioeconomic status, arguably because all study participants initially had a similar high educational level. During 30 years of follow-up, however, considerable changes may have occurred in family income and marital status among the study participants [10].

Elucidation of potential pathogenic mechanisms behind higher mortality rates with high consumption of non-fermented milk, irrespective of fat content, is not within the scope of our present analysis. There are, however, previous suggestions of higher oxidative stress and inflammation from the galactose component of milk sugar (lactose) [4] and that milk components as branched-chain amino acids as well as milk-derived exosomes can stimulate the mammalian target of rapamycin (mTOR) pathway, leading to earlier development of age-related disorders and premature mortality [11,12].

In conclusion, increasing non-fermented milk intake, irrespective of fat content, is related to all-cause mortality in a dose-dependent pattern.

## Figures and Tables

**Table 1:** Baseline characteristics of the women in the Swedish Mammography Cohort.

	Categories of Total Milk Intake			
	<1 Glass/Day	1 to <2 Glasses/Day	2 To <3 Glasses/Day	≥3 Glasses/Day
	(<200 mL/Day)	(200–399 mL/Day)	(400–599 mL/Day)	(≥600 mL/Day)
*N*	16,926	23,438	15,461	5608
Age (years) at entry	53.2 (9.6)	54.0 (9.7)	54.1 (9.9)	52.8 (9.6)
Body mass index (kg/m^2^)	24.4 (3.9)	24.7 (3.8)	25.0 (4.0)	24.9 (4.2)
Height (m)	1.64 (0.06)	1.64 (0.06)	1.64 (0.06)	1.64 (0.06)
Total milk intake (mL/day)	17.3 (37.3)	201.6 (14.9)	400.2 (6.0)	676.8 (151.9)
Low fat (0.5%) milk intake (mL/day)	6.4 (24.2)	81.1 (99.4)	176.5 (198.8)	288.9 (345.5)
Medium fat (1.5%) milk intake (mL/day)	5.7 (23.6)	56.9 (91.0)	102.4 (174.8)	154.6 (292.1)
High fat (3%) milk intake (mL/day)	5.3 (19.6)	63.7 (93.5)	121.1 (183.8)	233.8 (338.1)
Soured milk/yogurt, mean (SD)	102.4 (117.2)	97.6 (101.2)	93.3 (105.1)	87.0 (113.1)
Cheese, gram/day, mean (SD)	26.8 (21.3)	26.2 (19.6)	26.8 (19.9)	27.7 (22.0)
Energy intake (kcal/day)	1412 (433)	1535 (414)	1707 (435)	1965 (527)
Calcium intake (mg/day)	716 (221)	895 (192)	1043 (202)	1194 (245)
Education (≤9 years) *n* (%)	13,094 (78.9)	18,497 (80.3)	12,388 (81.6)	4377 (79.3)
Education (10–12 years) *n* (%)	1259 (7.6)	1576 (6.8)	972 (6.4)	368 (6.7)
Education (>12 years) *n* (%)	868 (5.2)	1106 (4.8)	594 (3.9)	254 (4.6)
Education (other) *n* (%)	1375 (8.3)	1867 (8.1)	1232 (8.1)	519 (9.4)
Married/cohabiting *n* (%)	12,814 (76.4)	17,932 (77.2)	11,565 (75.5)	4117 (74.1)
0 Charlson comorbidities *n* (%)	15,745 (93.0)	21,884 (93.4)	14,385 (93.0)	5146 (91.8)
1 Charlson comorbidity *n* (%)	1048 (6.2)	1350 (5.8)	927 (6.0)	390 (7.0)
2 or more Charlson comorbidities *n* (%)	133 (0.8)	204 (0.9)	149 (1.0)	72 (1.3)

**Table 2:** Hazard ratios (HRs) and 95% confidence intervals (CI) of total mortality, according to consumption of type of non-fermented milk in women from the Swedish Mammography Cohort with the exposure time-updated as cumulative averages.

**Low Fat Milk (0.5% fat)**					
**(A) Restriction to Those Consuming Less than One Serving Per Day of Other Types of Milk (1.5% or 3% fat)**					
	**Milk Intake Category (0.5% fat)**				
	<200 mL/day	200–399 mL/day	400–599 mL/day	≥600 mL/day	Per 200 mL/day (continuous)
N deaths	7650	3119	1804	598	13,171
Person-years at risk	534,282	215,813	118,779	39,127	908,001
Death rate (95% CI)	14.3	14.5	15.2	15.3	14.5
HR (95% CI), model 1	1.00 (1.00–1.00)	1.15 (1.10–1.20)	1.55 (1.48–1.64)	1.90 (1.75–2.06)	1.14 (1.12–1.16)
HR (95% CI), model 2	1.00 (1.00–1.00)	1.11 (1.07–1.16)	1.39 (1.32–1.47)	1.71 (1.57–1.86)	1.12 (1.10–1.14)
**(B) Retain All Types of Milk Consumers in the Analysis (full Cohort; “Contaminated” Reference Category)**					
	**Milk Intake Category (0.5% fat)**				
	<200 mL/day	200–399 mL/day	400–599 mL/day	≥600 mL/day	Per 200 mL/day
N deaths	16,292	3518	2028	683	22,521
Person-years at risk	1,031,416	235,664	128,988	42,608	1,438,676
Death rate (95% CI)	15.8	14.9	15.7	16.0	15.7
HR (95% CI), model 1	1.00 (1.00–1.00)	0.92 (0.89–0.95)	1.19 (1.13–1.24)	1.38 (1.28–1.50)	0.99 (0.98–1.01)
HR (95% CI), model 2	1.00 (1.00–1.00)	0.96 (0.92–0.99)	1.14 (1.09–1.20)	1.34 (1.24–1.45)	1.02 (1.00–1.03)
HR (95% CI), model 3	1.00 (1.00–1.00)	0.98 (0.95–1.02)	1.19 (1.14–1.25)	1.41 (1.30–1.52)	1.02 (1.00–1.03)
**Medium Fat Milk (1.5% fat)**					
**(A) Restriction to Those Consuming less than One Serving Per day of Other Types of Milk (0.5% or 3% fat)**					
	**Milk Intake Category (1.5% fat)**				
	<200 mL/day	200–399 mL/day	400–599 mL/day	≥600 mL/day	Per 200 mL/day
N deaths	7650	2220	1177	357	11,404
Person-years at risk	534,282	135,782	65,636	20,109	755,809
Death rate (95% CI)	14.3	16.3	17.9	17.8	15.1
HR (95% CI), model 1	1.00 (1.00–1.00)	1.25 (1.19–1.31)	1.75 (1.64–1.86)	2.10 (1.89–2.34)	1.19 (1.16–1.21)
HR (95% CI), model 2	1.00 (1.00–1.00)	1.21 (1.15–1.27)	1.54 (1.44–1.64)	1.82 (1.63–2.04)	1.15 (1.13–1.18)
**(B) Retain all Types of Milk Consumers in the Analysis (Full Cohort; “Contaminated” Reference Category)**					
	**Milk Intake Category (1.5% fat)**				
	<200 mL/day	200–399 mL/day	400–599 mL/day	≥600 mL/day	Per 200 mL/day
N deaths	17,683	2926	1462	450	22,521
Person-years at risk	1,168,398	168,026	78,108	24,143	1,438,676
Death rate (95% CI)	15.1	17.4	18.7	18.6	15.7
HR (95% CI), model 1	1.00 (1.00–1.00)	0.93 (0.90–0.97)	1.22 (1.16–1.29)	1.47 (1.34–1.61)	0.98 (0.96–1.00)
HR (95% CI), model 2	1.00 (1.00–1.00)	0.97 (0.93–1.00)	1.17 (1.11–1.24)	1.38 (1.25–1.51)	1.00 (0.98–1.02)
HR (95% CI), model 3	1.00 (1.00–1.00)	0.96 (0.93–1.00)	1.19 (1.13–1.26)	1.38 (1.25–1.52)	0.99 (0.97–1.01)
**High Fat Milk (3% fat)**					
**(A) Restriction to Those Consuming Less than One Serving Per Day of Other Types of Milk (0.5% or 1.5% fat)**					
	**Milk Intake Category (3% fat)**				
	<200 mL/day	200–399 mL/day	400–599 mL/day	≥600 mL/day	Per 200 mL/day
N deaths	7650	2464	1402	518	12,034
Person-years at risk	534,282	144,657	78,282	30,487	787,708
Death rate (95% CI)	14.3	17.0	17.9	17.0	15.3
HR (95% CI), model 1	1.00 (1.00–1.00)	1.45 (1.39–1.52)	1.99 (1.88–2.11)	2.52 (2.30–2.76)	1.31 (1.29–1.34)
HR (95% CI), model 2	1.00 (1.00–1.00)	1.24 (1.18–1.30)	1.60 (1.50–1.70)	1.95 (1.77–2.15)	1.20 (1.18–1.23)
**(B) Retain All Types of Milk Consumers in the Analysis (Full Cohort; “Contaminated” Reference Category)**					
	**Milk Intake Category (3% fat)**				
	<200 mL/day	200–399 mL/day	400–599 mL/day	≥600 mL/day	Per 200 mL/day
N deaths	17,183	2977	1725	636	22,521
Person-years at risk	1,142,947	166,735	92,019	36,975	1,438,676
Death rate (95% CI)	15.0	17.9	18.7	17.2	15.7
HR (95% CI), model 1	1.00 (1.00–1.00)	1.11 (1.06–1.15)	1.40 (1.33–1.47)	1.61 (1.49–1.74)	1.11 (1.09–1.13)
HR (95% CI), model 2	1.00 (1.00–1.00)	1.02 (0.98–1.06)	1.21 (1.15–1.27)	1.40 (1.29–1.52)	1.05 (1.04–1.07)
HR (95% CI), model 3	1.00 (1.00–1.00)	1.05 (1.01–1.10)	1.27 (1.21–1.34)	1.47 (1.35–1.60)	1.07 (1.05–1.08)
**Total Milk Intake**	**Milk Intake Category (any fat content)**				
	<200 mL/day	200–399 mL/day	400–599 mL/day	≥600 mL/day	Per 200 mL/day
N deaths	8332	8381	4296	1512	22,521
Person-years at risk	573,831	515,685	258,732	90,429	1,438,676
Death rate (95% CI)	14.5	16.3	16.6	16.7	15.7
HR (95% CI), model 1	1.00 (1.00–1.00)	1.28 (1.24–1.32)	1.81 (1.74–1.87)	2.18 (2.06–2.30)	1.18 (1.16–1.19)
HR (95% CI), model 2	1.00 (1.00–1.00)	1.18 (1.14–1.22)	1.55 (1.49–1.61)	1.83 (1.72–1.94)	1.13 (1.11–1.15)
HR (95% CI), model 3	1.00 (1.00–1.00)	1.19 (1.15–1.23)	1.58 (1.51–1.64)	1.84 (1.74–1.96)	1.13 (1.11–1.14)

Model 1 Adjusted for age; Model 2 Adjusted for age, body mass index, height, total energy intake, total alcohol intake, healthy dietary pattern (all continuous), calcium (yes/no) and vitamin D (yes/no) supplementation, ever use of cortisone (yes/no), educational level (≤9, 10–12, >12 years, other), living alone (yes/no), physical activity level estimated as metabolic equivalents (continuous), smoking status (never, former, current), estrogen replacement therapy (yes/no), nulliparity (yes/no), and Charlson’s comorbidity index (continuous); Model 3. Adjusted for covariates in Model 2 and additionally for the intakes of the other milk fat subtypes (e.g., low fat milk is thus adjusted for intake of 1.5% fat milk and 3% fat milk). Model 3 for total milk intake included covariates in Model 2 and additionally total intake of saturated fat.

**Table 3:** Age-adjusted differences in milk intake (mL/day), overall (column Total) and by fat content (columns 0.5% fat, 1.5% fat, and 3% fat, respectively), between categories of educational levels and marital status at baseline 1987–1990.

	Type of Milk by Fat Content (Differences in mL/Day)			
	Total	0.5% Fat	1.5% Fat	3% Fat
Education				
≤9 years	reference	reference	reference	reference
10–12 years	−13.0 (−19.5 to −6.6)	−0.6 (−6.3 to 5.1)	−1.1 (−5.7 to 3.5)	−9.9 (−15.1 to −4.7)
>12 years	−19.4 (−27.1 to −11.6)	−3.9 (−10.8 to 2.9)	7.9 (2.4 to 13.5)	−20.3 (−26.5 to −14.0)
Other	2.8 (−3.1 to 8.8)	−3.2 (−8.4 to 2.0)	7.0 (2.8 to 11.3)	−0.6 (−5.4 to 4.1)
Marital status				
Married or cohabiting	reference	reference	reference	reference
Unmarried or living alone	12.7 (8.9 to 16.6)	−13.0 (−16.4 to −9.7)	10.1 (7.4 to 12.8)	13.3 (10.3 to 16.4)
